# Variable Incidence of *Spiroplasma* Infections in Natural Populations of *Drosophila* Species

**DOI:** 10.1371/journal.pone.0005703

**Published:** 2009-05-28

**Authors:** Thomas Watts, Tamara S. Haselkorn, Nancy A. Moran, Therese A. Markow

**Affiliations:** Department of Ecology and Evolutionary Biology, University of Arizona, Tucson, Arizona, United States of America; Aarhus University, Denmark

## Abstract

*Spiroplasma* is widespread as a heritable bacterial symbiont in insects and some other invertebrates, in which it sometimes acts as a male-killer and causes female-biased sex ratios in hosts. Besides *Wolbachia,* it is the only heritable bacterium known from *Drosophila*, having been found in 16 of over 200 *Drosophila* species screened, based on samples of one or few individuals per species. To assess the extent to which *Spiroplasma* infection varies within and among species of *Drosophila*, intensive sampling consisting of 50–281 individuals per species was conducted for natural populations of 19 *Drosophila* species. Infection rates varied among species and among populations of the same species, and 12 of 19 species tested negative for all individuals. *Spiroplasma* infection never was fixed, and the highest infection rates were 60% in certain populations of *D. hydei* and 85% in certain populations of *D. mojavensis*. In infected species, infection rates were similar for males and females, indicating that these *Spiroplasma* infections do not confer a strong male-killing effect. These findings suggest that *Spiroplasma* has other effects on hosts that allow it to persist, and that environmental or host variation affects transmission or persistence leading to differences among populations in infection frequencies.

## Introduction

Based on recent molecular surveys, heritable bacterial symbionts are widespread in arthropods, but, in most cases, their effects on hosts are unknown (e.g., [1], [2]. *Drosophila* species harbor only two types of heritable bacterial endosymbionts [3], [4]. The most widely studied, and the most common, is *Wolbachia* [5], [3]. The other heritable bacterial endosymbiont in *Drosophila* is *Spiroplasma*, now reported in a total of 16 species [6], [7], [8], [9], [3], [10] and, curiously, rarely found to coinfect with *Wolbachia*. In some *Drosophila* species, *Spiroplasma* causes male-killing [11], [12], [13], [8], while in others it does not [14], [3], [11]. *Spiroplasma* has been studied far less than *Wolbachia*, and factors underlying its distribution among and within *Drosophila* species are unknown.

Factors potentially affecting endosymbiont infection prevalence include the transmission fidelity of the bacteria and its effects on host fitness. Vertical transmission can exhibit high fidelity as evidenced by the decades-long persistence of *Spiroplasma*-positive strains of *D. hydei* and *D. aldrichi* in the *Drosophila* Species Stock Center [3]. Experimental studies show that temperature affects fidelity of maternal inheritance of *Spiroplasma* in *Drosophila* hosts, suggesting that infections may be influenced by climate or microhabitat [15], [16], [17]. Condition-dependent effects on host fitness or reproduction also can influence infection frequencies. Male-killing endosymbionts can be favored in conditions where female offspring benefit from reduced competition from their male siblings [18]. In other insects, heritable symbionts often provide defenses against temperature stress or natural enemies, leading to fitness advantages of infected lineages [19].

Field surveys from wild populations of *D. hydei* revealed infection rates of 23–66% of females, the highest levels yet reported for any *Drosophila* [14]. In contrast, infection of wild *D. willistoni* and *D. nebulosa* by male-killing *Spiroplasma* ranged from 1–6%, varying seasonally [6]. These earlier studies suggest interspecific differences in infection rates, but limitations in sampling design or extent prevent inference regarding infection patterns or dynamics. Rates of infection by male-killing compared to non-male-killing *Spiroplasma* within and among different *Drosophila* species need to be examined before the basis for infection and its persistence can be understood.

**Figure 1:**
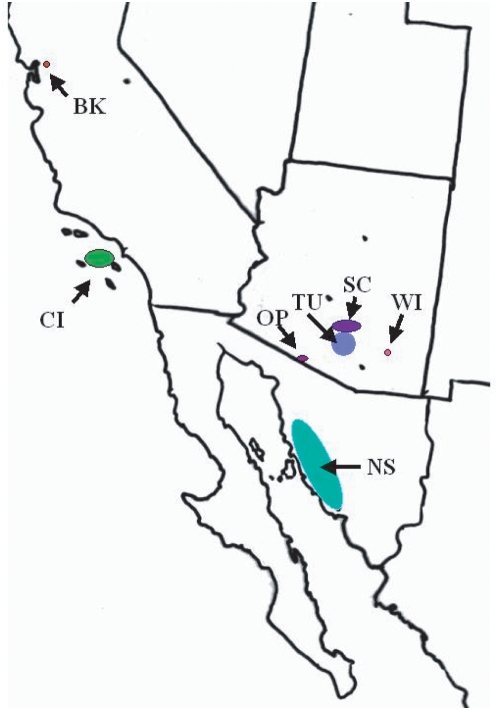
Collection localities for Drosophila. BK = Berkeley, CA, CI = Catalina Island, CA, OP = Organ Pipe Cactus Nat'l Mon, AZ, TU = Tucson, AZ, SC = Santa Catalina Mts, AZ, WI = Willcox, AZ, NS = Northwestern Sonora, MX.

*Drosophila* species vary widely in their geographic distributions and ecologies [20]. The natural abundance of multiple *Drosophila* species at any given locality provides an opportunity to perform larger-scale screening in wild populations and to address questions about the ecological and evolutionary dynamics of *Spiroplasma* infections. We examined infection status in wild-caught females and males of 19 *Drosophila* species from localities (Figure 1) in the southwestern United States and northwestern Mexico in order to (1) ask how the incidence of infected flies varies in nature and (2) assess the sex ratio of infected flies in order to detect evidence of male killing infections. Our screen employed PCR primers universal for *Spiroplasma*, rather than those used to target male-killing strains, resulting in as complete detection as possible. Furthermore, a greater depth of sampling within each species allowed us to detect *Spiroplasma* infections at low frequencies.

## Materials and Methods

Flies were collected at the localities shown in Figure 1 either directly from cactus (*D. mojavensis),* cave walls (*D. macroptera, D. grisea*), or from mushroom (*D. tenebrosa*) and banana baits (other species) (Table 1). Live flies were keyed to species and sex, maintained on species-appropriate culture medium for several days, and then frozen.

**Table 1:** *Drosophila* species screened, dates and locations of collection.

Subgenus	Species	Collection Site	Date	Zone
*Drosophila*	D. aldrichi	Tucson, AZ	2006–2007	Desert
	*D. arizonae*	Tucson, AZ	2006–2007	Desert
		NW Sonora, Mex.	2006–2007	Desert
		Organ Pipe Natl. Mon. AZ	2007	Desert
	*D. carbonaria*	Tucson, AZ	2006–2008	Desert
	*D. grisea*	Catalina Mts. AZ	2007–2008	Montane
	*D. hamatofila*	Catalina Isl., CA	2002,2006–2007	Coastal
	*D. hydei*	Tucson, AZ	2006–2008	Desert
		NW Sonora, Mex	2006–2008	Desert
		Willcox, AZ	2007	Prairie
	*D. Immigrans*	Berkeley, CA	2007–2008	Temperate
		Tucson, AZ	2008	Desert
	*D. macroptera*	Catalina Mts., AZ	2007	Montane
	*D. mettleri*	Catalina Isl., CA	2002, 2006–2007	Coastal
		Tucson, AZ	2006–2007	Desert
		NW Sonora, Mex	2006–2007	Desert
	*D. mojavensis*	Catalina Isl., CA	2007	Coastal
		Organ Pipe Natl. Mon. AZ	2007	Desert
		NW Sonora, Mex.	2006–2007	Desert
	*D. nigrospiracula*	Organ Pipe Natl. Mon., AZ	2007	Desert
		Tucson, AZ	2006–2007	Desert
		NW Sonora, Mex.	2008	Desert
	*D. pachea*	Organ Pipe Natl. Mon., AZ	2007	Desert
		Tucson, AZ	2007	Desert
		NW Sonora, Mex.	2007	Desert
	*D. rubrifrons*	Catalina Mts., AZ	2007	Montane
	*D. tenebrosa*	Catalina Mts., AZ	2007	Montane
	*D. wheeleri*	Catalina Isl., CA	2002, 2006	Coastal
				
*Sophophora*	*D. simulans*	Catalina Isl., CA	2006–2007	Coastal
		Tucson, AZ	2006–2008	Desert
		NW Sonora, Mex.	2007–2008	Desert
		Catalina Mts., AZ	2008	Montane
	*D. melanogaster*	NW Sonora, Mex.	2007–2008	Desert
		Tucson, AZ	2006–2007	Desert
	*D. pseudoobscura*	Catalina Isl., CA	2006	Coastal
		Tucson, AZ	2006–2008	Desert
		NW Sonora, Mex.	2007	Desert
		Catalina Mts., AZ	2008	Montane
*Dorsilopha*	*D. busckii*	Berkeley, CA	2007–2008	Coastal
		Tucson, AZ	2008	Desert

DNA extraction from individual flies was carried out as previously described [3]. Briefly, whole flies were extracted with the single-fly squish prep protocol [21]. PCR screens for *Spiroplasma* were based on amplification of an approximately 410 base pair fragment of bacterial 16S rDNA using the spiroplasma-diagnostic primers 23f (5′-CTCAGGATGAACGCTGGCGGCAT-3′) and TKSSsp (TAGCCGTGGCTTTCTGGTAA [22]) and a touchdown thermal cycler program [3]. The initial screening PCR volume was 10 ul. These primers are expected to amplify almost all *Spiroplasma* strains and would amplify male-killing and non-male killing strains known from insects, based on comparison to sequence databases. The primers also have the potential to amplify some other groups of Bacteria.

To verify the identify of positive samples as *Spiroplasma*, each was re-amplified at larger volume (50 μl), and both strands were sequenced with an ABI 3700 at the University of Arizona's Genomics Analysis & Technology Core facility. As a check for DNA quality, all samples were screened for a fragment of mitochondrial cytochrome c oxidase I gene (COI) using primers HCO and LCO with an annealing temperature of 45°C [3]. Only samples that gave positive amplifications for COI were included in the survey. Sequences were edited and aligned using Mega 3.1 [23] and identified using Blastn [24] to query the nr database at GenBank.

## Results

**Figure 2:**
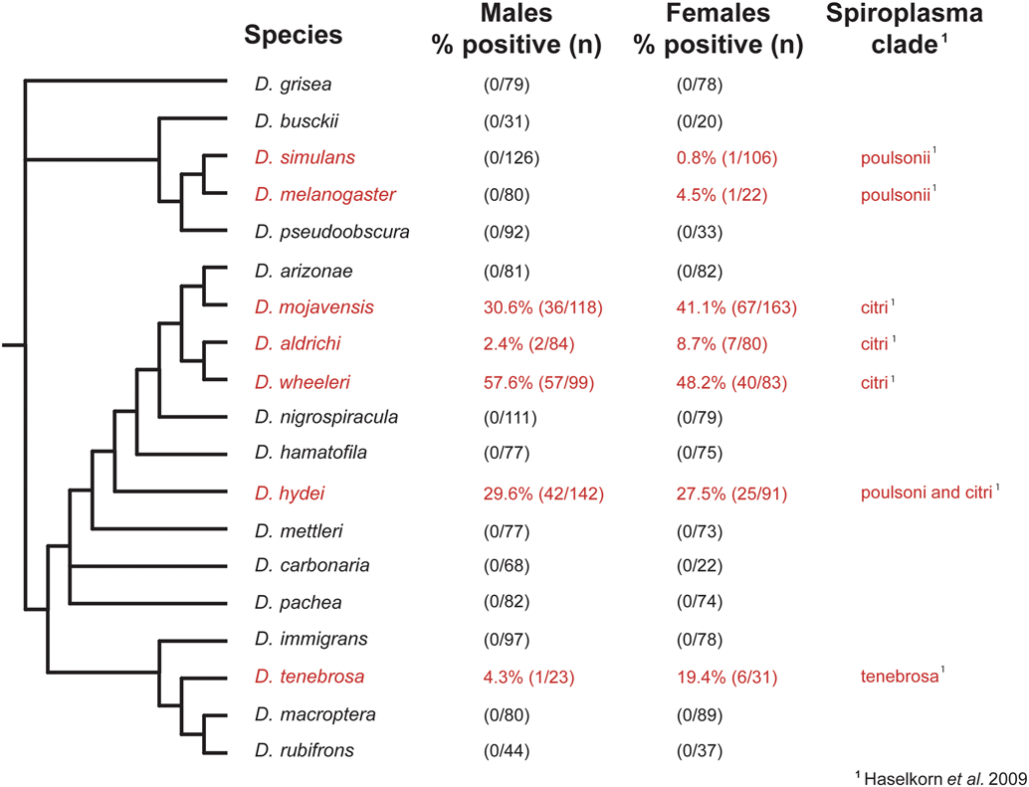
Frequency of *Spiroplasma* infection in wild-caught *Drosophila.* The phylogenetic relationships of *Drosophila* are represented as a cladogram based on Markow & O'Grady [20] *Spiroplasma*-infected species are colored in red.

Of 19 *Drosophila* species screened, *Spiroplasma* was found in seven **(** Figure 2). Infection incidence ranged from under 1% in *D. simulans* and *D. melanogaster* to an average of 37% in *D. mojavensis*. Some species are relatively rare in nature, such that fewer individuals were collected and screened. Sex differences in infection were not significant, although in the case of *D. aldrichi* the excess of infected females approached significance (X^2^ = 3.20, 0.10>p>0.05). In *D. hydei,* more than one *Spiroplasma* strain was distinguishable based upon 16S rDNA sequence, although no co-infections with distinct symbionts were observed within the same host [10], [3].

For two species, sampling permitted comparisons between localities (Table 2). For *D. hydei*, the proportion of infected flies was several times higher for samples from Willcox, Arizona than for samples from Sonora. For *D. mojavensis*, infection rate was higher at Santa Catalina Island than at Organ Pipe National Monument.

**Table 2:** Frequency of infection in populations of *D hydei* and of *D mojavensis*

Species	Population	Males	Females
*D hydei*	Northwestern Sonora, MX	27.0% (34/126)	24.7% (19/77)
	Wilcox, AZ	60.0% (6/10)	60.0% (6/10)
*D mojavensis*	Organ Pipe National Monument, AZ	16.9% (13/77)	14.0% (12/86)
	Santa Catalina Island, CA	84.6% (22/26)	84.6% (55/65)

## Discussion

Our results represent the largest number of wild-caught insects screened to date for *Spiroplasma*. Over a third of the species screened showed *Spiroplasma* infection, though none of these species appeared to harbor a previously identified male-killing *Spiroplasma* strain. All of our positive samples were verified with sequencing. Although false negatives are possible (if our primers failed to amplify a novel strain), our screen would have detected known insect Spiroplasma strains, including male-killers and non-male-killers. A multi-locus sequence phylogenetic analysis of 69 of these Drosophila spiroplasmas revealed a large genetic diversity among *Spiroplasma* haplotypes. Based on this Bayesian phylogenetic analysis, the *Drosophila* spiroplasmas fall into four distinct, well-supported clades of the *Spiroplasma* phylogeny, with the most distantly related strain from the male-killing spiroplasmas having 14% sequence divergence at the 16S rDNA locus [10]. Furthermore, estimates of infection prevalence are likely to be conservative, as the sensitivity of our PCR screen may miss *Drosophila* with low *Spiroplasma* titer. Two infected species were in the subgenus Sophophora and five were in the subgenus *Drosophila*. Infection rates were considerably higher among infected species in the *Drosophila* subgenus compared to infected Sophophoran species. There was no pattern of infection related to geographic area.

**Figure 3:**
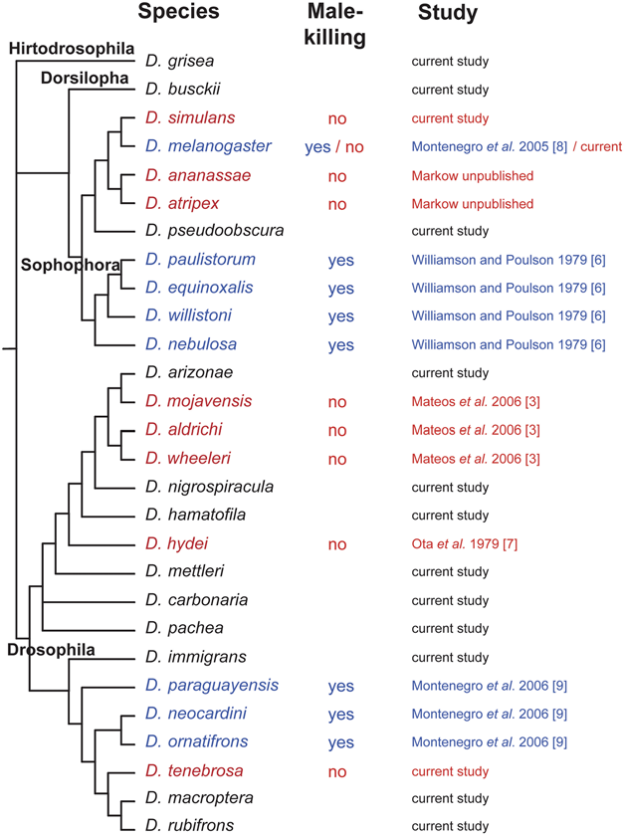
Distribution of male-killing and non-male-killing Spiroplasma in natural populations of *Drosophila* species surveyed to date. The phylogenetic relationships of *Drosophila* are represented as a cladogram based on Markow & O'Grady [20] Non-male-killing *Spiroplasma*-infected species are colored in red and male-killing *Spiroplasma*-infected species are in blue.

By screening both sexes for each species, we obtained indications as to whether *Spiroplasma* is acting as a male-killer, as known for some *Drosophila* [8]. In addition, each screening reaction had a positive control, the male-killing *Spiroplasma* infecting D. melanogaster [11]. Our primers were able to detect spiroplasmas up to 14% sequence divergent from the male-killing strain at the 16S rDNA locus. Other than for *D. simulans* and *D. melanogaster*, in which the infection frequency was under 1%, both sexes of infected species were found to be *Spiroplasma*-positive, indicating the absence of a strong male-killing phenotypes. Nor was the *Spiroplasma* found in the *D. melanogaster* female a male-killer, as the strain was established in culture and yielded infected flies of both sexes. Thus the male-killing effect does not appear to be a general explanation for the presence of *Spiroplasma* in these insects. Furthermore, as the number of *Drosophila* species found to be infected with *Spiroplasma* grows, the male-killing phenotype continues to be restricted to particular lineages, primarily the subgenus *Sophophora* and in the tripunctata radiation in the subgenus *Drosophila* (Figure 3).

Host genotype clearly influences the distribution of *Spiroplasma* within as well as among *Drosophila* species. For example, *D. willistoni* shows intraspecific variation affecting *Spiroplasma* transmission [13], [25], [6]. Infection rates for natural populations of *D. hydei* in our study are similar to those reported by Kageyama *et al.* [14] reflecting a consistent pattern for this species from different global regions. *Drosophila aldrichi*, in which fewer than 10% of individuals were spiroplasma-positive, clearly shows a lower frequency of infected individuals of both sexes relative to *D. hydei*. In *D. simulans,* and *D*. *melanogaster* the infection level is even lower (Figure 2.) In contrast to the *Wolbachia* infections in *D. innubila* [26], infections with non-male-killing *Spiroplasma* appear to be more, as opposed to less, frequent than infections with male-killing types.

Though multiple factors likely affect spiroplasma prevalence, the fidelity of vertical transmission may play a role. Temperature affects maternal transmission of *Spiroplasma* in *D. melanogaster* and *D. nebulosa* [15], [17] and in *D. hydei* [16]. Similarly, field conditions including temperature influence maternal transmission efficiency of *Wolbachia* in *Drosophila* hosts [27], [28], [29], [30]. In our study, both *D. mojavensis* and *D. hydei* were collected from two locations and each showed a lower infection rate at the hotter site (Table 2). Transmission efficiency may be decreased at low temperatures, as shown experimentally for *D. hydei* [16], and also at the extreme high temperatures that occur at some desert localities sampled in our survey.

The variation in natural infection rates reported here, both among and within species, indicates a dynamic system in which infection, fitness effects and persistence of spiroplasmas in *Drosophila* are dependent upon the interplay of symbiont and host genotype and local environmental conditions. Given the ease of rearing and manipulating a range of evolutionarily, ecologically and genetically defined *Drosophila* species, our opportunities to disentangle and understand the roles of these factors are unparalleled.
